# Biologic plating of unstable distal radial fractures

**DOI:** 10.1186/s12891-018-2046-2

**Published:** 2018-04-14

**Authors:** Jae-Man Kwak, Gu-Hee Jung

**Affiliations:** 1 Department of Orthopaedic Surgery, Asan Medical Center, University of Ulsan, Seoul, Republic of Korea; 2 Department of orthopaedic surgery, Gyeongsang national university, college of medicine and Gyeongsang national university Changwon hospital, 555 Samjungja-Dong, Changwon-si, 642-160 Republic of Korea

**Keywords:** Distal radial fracture, Bridging plate, PQ-sparing technique

## Abstract

**Background:**

Volar locking plating through the flexor carpi radialis is a well-established technique for treating unstable distal radial fractures, with few reported complications. In certain circumstances, including metaphyseal comminuted fractures, bridge plating through a pronator quadratus (PQ)-sparing approach may be required to preserve the soft tissue envelope. This study describes our prospective experience with bridge plating through indirect reduction.

**Methods:**

Thirty-three wrists (four 23A2, six 23A3, 15 23C1, and eight 23C2) underwent bridge plating through a PQ-sparing approach with indirect reduction from June 2006 to December 2010. Mean patient age was 56.8 years (range, 25–83 years), and the mean follow-up period was 47.5 months (range, 36–84 months). Changes in radiologic parameters (volar tilt, radial inclination, radial length, and ulnar variance) were analyzed, and functional results at final follow-up were evaluated by measuring the Modified Mayo Wrist Score (MMWS) and Modified Gartland-Werley Score (MGWS).

**Results:**

All wrists achieved bone healing without significant complications after a single operation. At final follow-up, radial length was restored from an average of 3.7 mm to 11.0 mm, as were radial inclination, from 16.4° to 22.5°, and volar tilt, from − 9.1° to 5.5°. However, radial length was overcorrected in three wrists, and two experienced residual dorsal tilt. Excellent and good results on the MGWS were achieved in 30 wrists (90.9%). The average MMWS outcome was 92.6 (range, 75–100).

**Conclusion:**

Our experience with bridge plating was similar to that previously reported in the earlier publications. Compared with the conventional technique, bridge plating through a PQ-sparing approach may help in managing metaphyseal comminuted fractures of both cortices with a reduced radio-ulnar index.

## Background

The increasing incidence of complex fracture patterns after high-energy trauma and the demand for early return to activities of daily living have increased the number of patients requiring operative intervention to treat distal radial fractures. Fractures may be reduced directly and fixed with volar locking plates [1–3]. This method has also yielded lower complication rates, ranging from 7% to 24%, than other treatment modalities [4]. The development of an internal fixator with locking plate technology has increased the rate of biologic fixation through minimally invasive plate osteosynthesis (MIPO, bridge plating). This method preserves soft tissue around the fracture site and prevents surgical morbidities of direct reduction in treating comminuted fractures of the lower limbs [5, 6]. Less is known, however, about the results of bridge plating for distal radial fractures [7–9].

Many factors influence the management of distal radial fractures, including the low complication rate of the open method, lesion size, and the easy manipulation and restoration of anatomic continuity using closed maneuvers. In addition, the postoperative radiologic and clinical outcomes of MIPO and conventional plating for distal radial fractures did not differ significantly [9]. The only advantages of MIPO were reported to be the preservation of the pronator quadratus (PQ) muscle and cosmesis, suggesting that conventional plating should be preferred to MIPO, provided that adequate reduction and sufficient fixation could be addressed [7–9]. Therefore, the clinical relevance of MIPO for distal radial fracture remains unclear.

Because MIPO involves indirect reduction through separate incisions, there are technical issues involved, with optimal results depending on anatomic restoration of the articular surface and extra-articular realignment [6]. However, few reports have assessed the indirect reduction technique involving MIPO for distal radial fractures [10]. This retrospective analysis of prospectively enrolled patients describes our experiences with bridge plating of distal radial fractures using a PQ-sparing approach, including the reduction technique, focusing on the usefulness and suggested indications of this method.

## Methods

The study protocol was approved by the ethics committee of our institution. Consecutive subjects with unstable distal radial fractures were enrolled prospectively from June 2006 to December 2010, and the results of bridge plating using a PQ-sparing approach were analyzed retrospectively. All patients with distal radial fracture were initially managed in the emergency room with closed reduction and splinting. This study excluded patients aged < 18 years, those with concomitant upper extremity injuries (e.g., carpal bone injury, forearm fracture, elbow, and humeral fractures), patients with pathologic fractures, patients who had previously undergone surgical treatment of the same wrist, and those whose fractures were fixed with a 3.5 mm T-plate. The 2.4 mm volar distal radius plating system (LDRS®; Synthes, Oberdorf, Switzerland) was used in all cases of volar plate fixation. Each patient was followed up for more than 36 months after surgery.

The plain radiographs and CT scans of all patients were reviewed by two investigators using the Picture Archiving and Communication System (PACS), with their classifications verified according to the AO comprehensive classification system (AO/OTA) [11]. All subjects provided uniform informed consent that the choice of direct fixation or bridge plating through a PQ-sparing approach for each distal radial fracture would be determined intraoperatively based on several factors, including the quality of intraoperative reduction, an unexpected fracture line, and unknown causes.

### Intraoperative decision on bridge plating or direct fixation

**Fig. 1:**
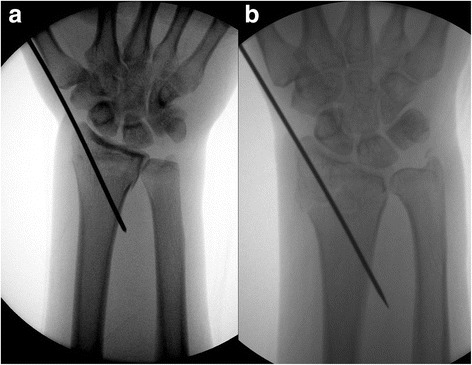
**a** After manual traction and manipulation of the injured wrist, the distal radial fracture was reduced with the restoration of radial length, with the reduction maintained by provisionally fixed K-wires from the radial styloid. **b** Although radial length was not restored, a provisional K-wire could be applied because of the distractive technique

Once surgery was indicated, all wrists underwent secondary evaluation by multidetector CT with multiplanar reformation, which has been used to determine the approach and implant for distal radial fractures [12, 13]. Radiologic parameters were measured by PACS, including changes in radial length and volar tilt, the site of joint involvement in the articular fractures, the length of the distal fragment, and the width of metaphyseal comminutions in both cortices. Once it was decided to use a single volar plate, manual traction was applied to the wrist, followed by manipulation under anesthesia with an image intensifier to restore radiologic parameters except for radial length. The fracture was provisionally fixed with K-wires from the radial styloid to maintain the reduction (Fig. 1). If reduction was acceptable, except in radial length, bridge plating through a PQ-sparing approach was chosen.

### Bridge plating through the PQ-sparing approach with indirect reduction

**Fig. 2:**
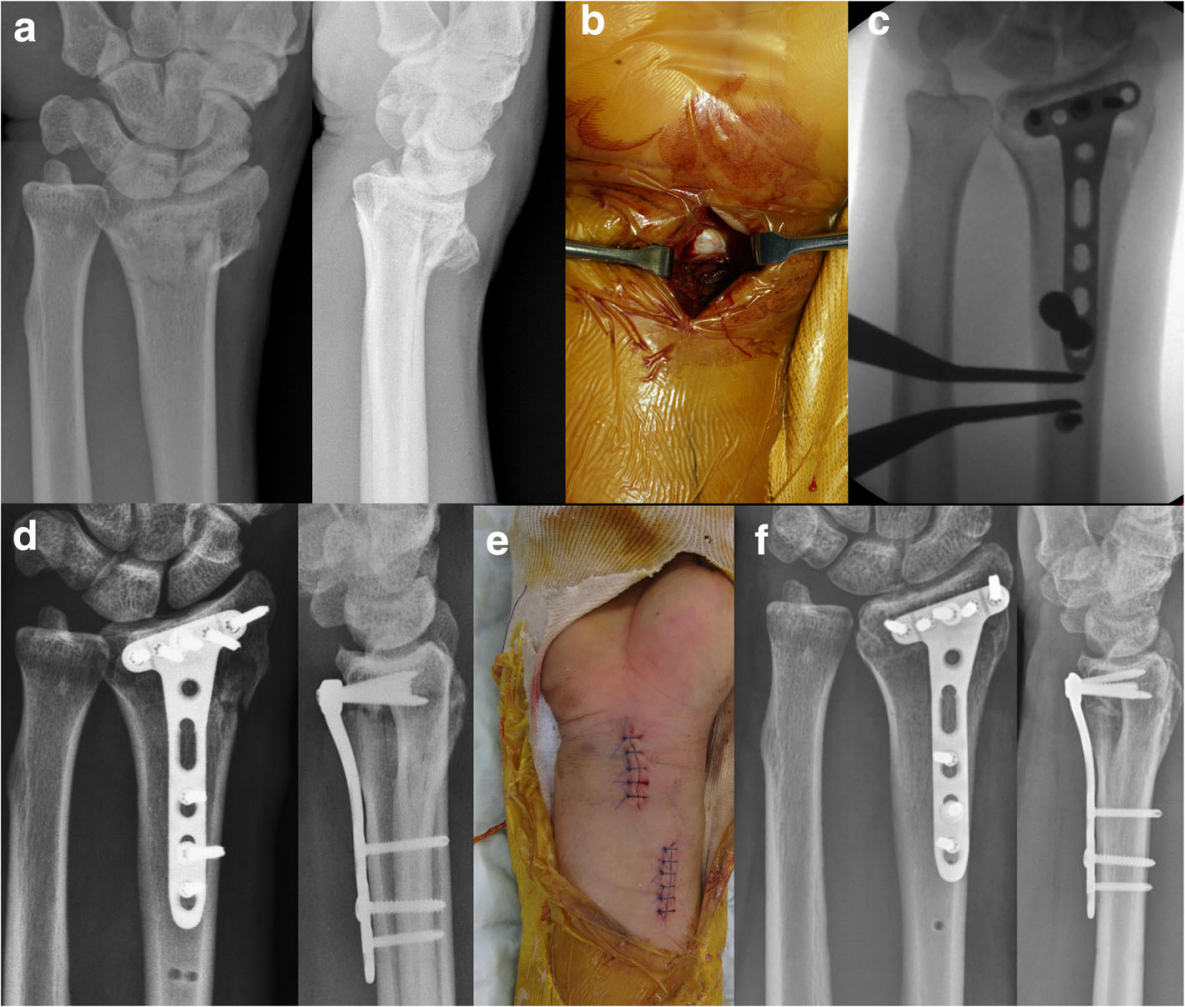
**a** Simple radiographs, showing a distal radial fracture with metaphyseal comminution and radial shortening. **b** A distal incision around the flexor carpi radialis tendon, exposing the distal margin of the PQ muscle. **c** Sliding of the plate through a PQ-sparing approach and the distractive technique to restore radial length using the spreader. **d** Postoperative radiographs showing good realignment of the distal radial fracture. **e** Two separated incisions. **f** After 16 weeks, the distal radial fracture was well healed without radial length shortening

**Fig. 3:**
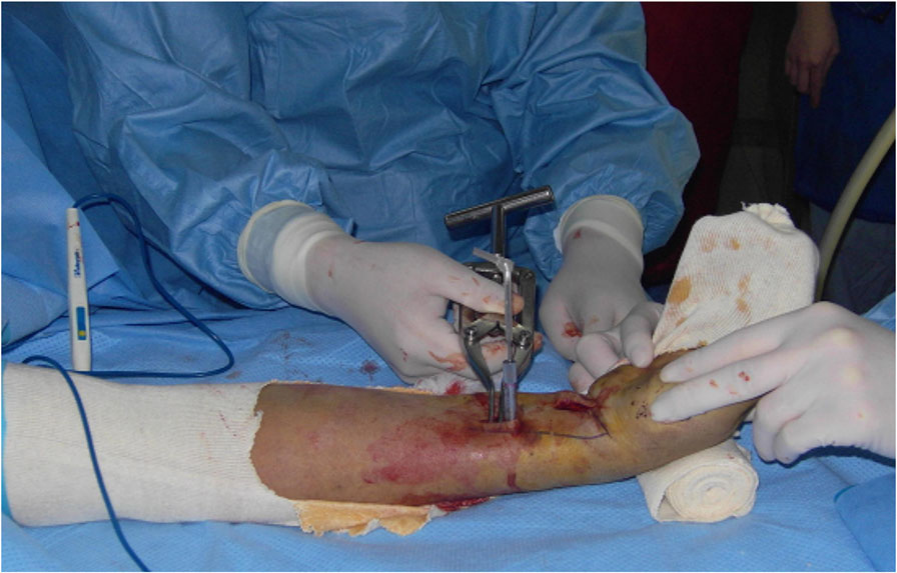
Application of a spreader for distraction using a screw outside the plate

Bridge plating through a PQ-sparing approach has been described in detail [10]. Once provisionally fixed, an image intensifier was used to archive the saved images of the contralateral wrist, which were used as reference guides to the restoration of radiologic parameters, including radial length and volar tilt, in the injured wrist. The tendon sheath was distally incised using a flexor carpi radialis (FCR) approach, allowing separate incisions for bridge plating, followed by exposure of the watershed line and distal margin of the PQ muscle and a proximal incision based on the length of the expected plate (Fig. 2). The plate was slid under the PQ muscle in a distal-to-proximal direction, and the optimal position of the plate on the distal radius was secured by inserting K-wires through the proximal and distal holes. After provisionally positioning the volar plate, radiologic parameters and conformity of the volar plate were thoroughly assessed. The plate was fixed with two or three screws in the distal hole and with a temporary K-wire in the proximal hole, thereby maintaining plate alignment. After securing the distal subchondral row with two or three screws, intraoperative fluoroscopy was performed to ensure that the distal locking screws were no more than 3–4 mm from the joint line and did not penetrate the joint surface. If the radial length was not restored, an additional screw was inserted proximally beyond the plate. Using a spreader, a distractive force was applied to restore radial length, comparing it with the saved image of the opposite wrist under the control of an image intensifier (Fig. 3, spreader). After verifying all the radiologic parameters, the empty holes on the plate were fixed and the wounds were closed.

### Outcome evaluations and statistical analysis

Wrists were evaluated radiologically by measuring the volar tilt (dorsal tilt is expressed as a negative value), radial inclination, and radial length (the distance between the most proximal point of the articular surface of the radius and the ulnar head, with positive values when the ulna is more distal than the radius and negative values when the ulna is more proximal than the radius) [14, 15]. Radiological parameters were measured preoperatively, immediately after surgery, and at the final follow-up visit using PACS, based on standard anteroposterior and lateral projection radiographs. These radiographs were compared with those of the non-injured wrist (Table 1). Activities of daily living at final follow-up were evaluated using the Modified Mayo Wrist Score (MMWS) and the Modified Gartland-Werley Score (MGWS) [16].

**Table 1:** Radiologic results after bridge plating of distal radius fracture

	Non-injured	Preop	Postop	Final FU
Volar tilt (°)	9.2 ± 4.3	− 9.1 ± 11.9	5.9 ± 11.0 (*p* = 0.0004)	5.5 ± 11.3 (*p* = 0.0004)
Radial inclination (°)	25.4 ± 3.9	16.4 ± 3.1	22.6 ± 8.1 (*p* = < 0.0001)	22.5 ± 9.1 (*p* = < 0.0001)
Radial length (mm)	9.8 ± 2.8	3.7 ± 4.9	11.2 ± 3.9 (*p* = < 0.0001)	11.0 ± 4.2 (*p* = 0.0004)
Radioulnar index (mm)		1.9 ± 2.1		−0.6 ± 2.6 (*p* = < 0.0001)

Data are mean ± standard deviation

*Preop* preoperative results, *Postop* postoperative results, *Final FU* final results at last follow-up

For statistical analysis, the radiologic results were expressed as mean and range, and their normal distribution was assessed using the Shapiro-Wilk test. Normally distributed parameters, including volar tilt and radial inclination, were compared using paired t-tests. Non-normally distributed parameters, including radial length and radio-ulnar index, were compared using Wilcoxon signed rank tests. A *P*-value < 0.05 was considered statistically significant. All statistical analyses were performed using SAS 9.2 software (SAS Institute, Cary, NC, USA).

## Results

This study enrolled 33 wrists of 28 patients, 12 men, and 16 men. Their mean age at surgery was 56.8 years (range, 25–83 years) and their mean follow-up duration was 47.5 months (range, 36–67 months). Two patients were lost during follow-up because of death (one suicide and one car accident). One patient underwent emergency surgery on the day of injury, but most underwent surgery about 7 days after injury. The causes of injury to the 28 patients included falling (*n* = 1), traffic accident (*n* = 12), industrial trauma (*n* = 2), and falling while standing (*n* = 13).

Of the 33 fractures, ten were type 23A, including four 23A2 and six 23A3 fractures, and 23 were type 23C, including 15 23C1 and eight 23C2 fractures. There were two open fractures, one Gustilo-Anderson (GA) type I and one GA type IIIA. The GA type I fracture was treated as a closed fracture on the day of the injury, whereas the type IIIA fracture underwent emergency surgery and application of a spanning external fixator because the distal ulnar head was protruding through the open wound.

Of the 33 fractures enrolled, none required intraoperative conversion to direct open reduction. Twenty-six patients (31 fractures) were managed with a single-stage operation, which included a surgical delay for conditioning the soft tissue around the injured wrist. However, the remaining two patients (two fractures) underwent staged definitive fixation as soon as the status of the soft tissue permitted. The average time from injury to surgery was 5.8 days (range, 0–16 days). One patient with an open fracture was initially treated with emergency irrigation and debridement in the operating room, followed 16 days later by definitive fixation, after recovery of the soft tissue envelope. The other patient presented with a crush injury, which resulted in comminuted fractures of the distal radius and ulna with severe swelling. This patient was initially treated with plate fixation of the ulna and application of the spanning external fixator. The time interval from the first operation to definitive fixation was 7 days. The mean operative time for all bridge plating procedures was 61 min (range, 33–98 min), and all 33 fractures were united without secondary procedure to promote bone healing.

Despite closed maneuvers and a surgical delay of more than 2 weeks in four patients, extra-articular realignment was achieved using the above technique, with all articular fractures showing restoration of joint incongruity to < 2 mm and significant restoration of all radiologic parameters (Table 1) sufficient for statistical significance. The radio-ulnar index (RUI) [14] was changed to a negative value by distraction using a spreader. Radiographs taken at the final visit revealed that the radiologic parameters were well maintained, except for slight losses of radial length and radial inclination in some wrists. Of the 23 wrists with articular fractures, 15 23C1 and eight 23C2, all but one with a C2 fracture had articular gaps < 2 mm. The patient with significant articular incongruity did not wish to undergo a second operation because of associated injury to the head and abdomen.

At final evaluation, all patients had achieved nearly full finger range of motion; that is, they were able to touch their palms with the fingertips and to fully extend their fingers to a neutral position. In addition, all patients achieved wrist flexion, as assessed by a goniometer, having > 90% motion compared with the uninjured wrist [17]. The average MMWS score was 92.6 points (range, 75–100 points), and 30 wrists (90.9%) showed excellent and good MGWS results. Overall outcomes were excellent in 15 patients, good in 10, and fair in three. None of these patients had residual wrist pain or sleep disturbance due to pain, and all were able to perform daily activities without restriction. During follow-up, however, three patients with excessive radial length experienced mild pain in the distal radio-ulnar joint at the first outpatient follow-up visit. Compared with the average RUI, the RUIs differed after surgery in these patients by 4.0, 4.0 and 5.0 mm, differences that were significant. Pain in all three of these patients resolved within 6 months.

None of these patients experienced deep infections or wound complications that required additional surgery. No patient complained of plate-related symptoms or underwent plate removal [18]. Five patients developed postoperative complications related to indirect reduction (Table 2). Two had insufficient reduction of the distal articular angle that was not fully restored to volar tilt by indirect methods, whereas three experienced transient pain in the distal radio-ulnar joint due to radial length overcorrection. None of these patients, however, experienced other conventional complications of volar plating, such as extensor tendon rupture, injury of the median nerve, or loosening.

**Table 2:** Complications related with bridge plating through pronator-sparing approach in distal radius fractures

Details	Cases No.
Indirect reduction	
intraoperative conversion	–
symptomatic over-correction of radial length	3
insufficient correction of volar tilt	2
Volar plate fixation	
Intra-articular screw placement	–
loosening of screw	–
loss of reduction & fixation	–
deep infection	–
Tendon problems	
tendon irritation or rupture	–
FCR adhesion	–
Carpal tunnel syndrome	–
Reoperation	
bone graft	–
plate removal	–

complications from subsequent fracture collapse rather than from time of surgery

## Discussion

Use of a volar locking plate has advantages in treating unstable fractures of the distal radius, as it is accompanied by osteosynthesis. The volar locking plate has expanded the indications for operative treatment of these fractures and greatly improved their clinical and radiologic outcomes [1, 3]. However, despite the popularity of bridge plating to treat complex fractures of the lower limbs, only a few reports have described the use of this technique to treat distal radius fractures [7–10, 19–21]. Although bridge plating using a PQ-sparing approach may have the advantage of PQ muscle preservation, the use of small incisions can prevent acceptable fracture reduction [7, 8]. Sen MK et al. indicated that this technique should not be used if it compromises the adequacy of reduction or fixation of the distal radius and that it be performed only in carefully selected patients [8].

**Fig. 4:**
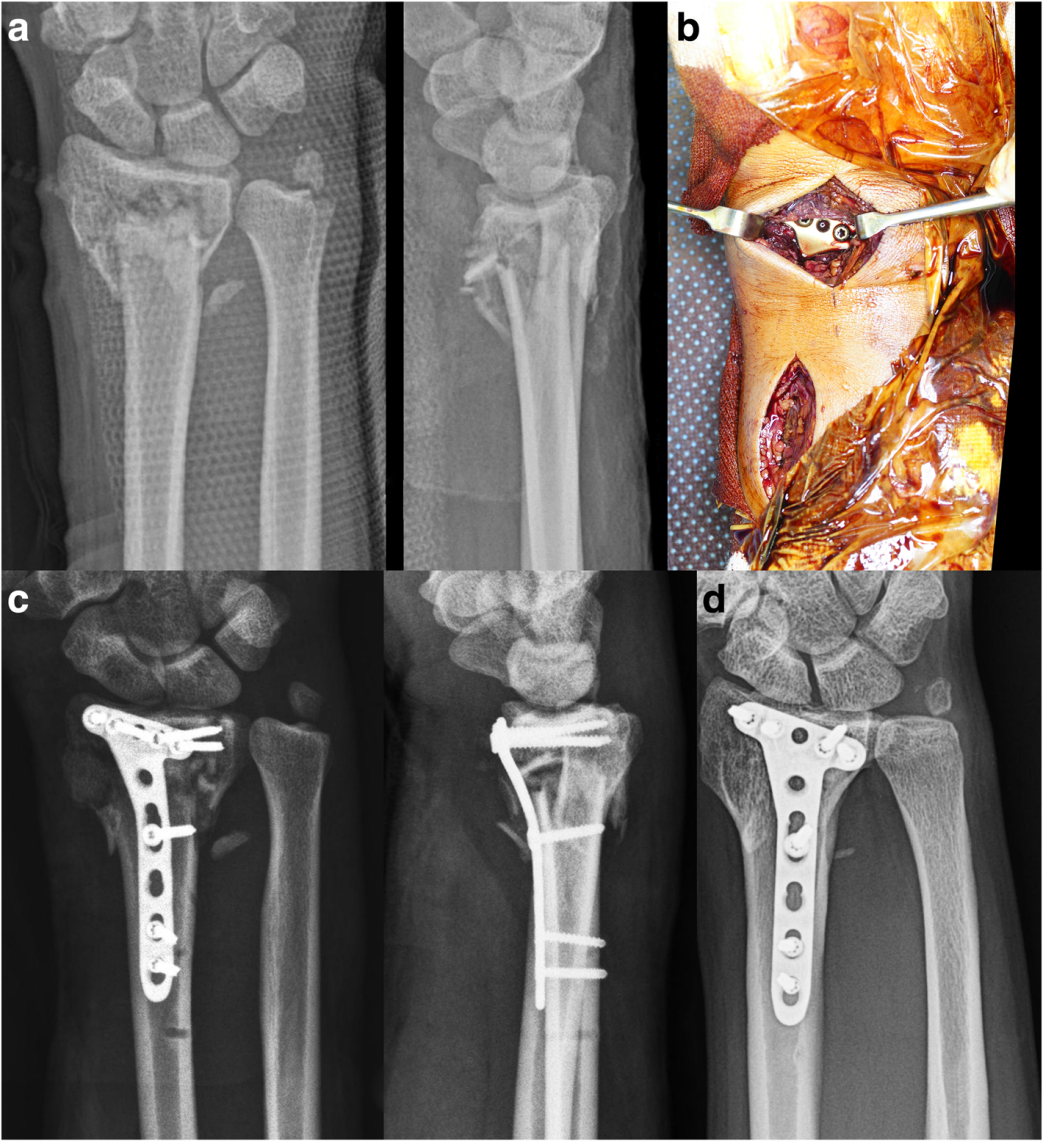
**a** A 34-year-old man fell from a height of 5 m. Simple radiographs showed metaphyseal comminution in both cortices. **b** Bridge plating through a PQ-sparing approach was performed with a distractive technique to fix a single volar plate. **c** Postoperative radiographs showing the restoration of radiologic parameters, including radial length, volar tilt, and radial inclination. **d** Radiographs after 24 months showing sound union without complications

Despite the proven benefits of direct reduction and anatomic fixation for distal radial fracture, the authors have adopted the concept of extra-articular realignment during prospective enrollment, especially for indirect reduction of metaphyseal fragments in accordance with MIPO technique. Compared with the concept of conventional anatomical reduction, extra-articular realignment is more focused on realignment of the injured limb by restoring the alignment index (length, rotation, and axis) rather than by anatomic reduction of the fracture fragments (Fig. 4). The reduction of distal radial fractures should focus on the restoration of radial length because most problems occurred in patients who achieved fair or poor restoration of radial length after conservative management [14]. Radial length was found to be automatically re-established upon restoration of anatomic continuity of the volar cortex through direct reduction [22]. Automatic restoration should not be expected, however, if a volar cortex is comminuted at the metadiaphyseal area or the fracture surfaces could not be opened, such as in the PQ-sparing approach, and an additional procedure might be needed to achieve the original length. A distractive technique using a spreader could be easily applied, similar to a femoral distractor [10]. Compared with the use of a large distractor or provisional external fixator to restore limb length [23], bridge plating with distractive technique has several benefits. That is, the reduction force could be controlled gradually by the surgeon and verified in the coronal and sagittal planes using an image intensifier in real time. Five wrists, however, experienced complications related to reduction obstacles, including two with undercorrection of the volar/residual dorsal tilt and three with overcorrection of radial length. Compared with the uninjured wrist, the average radial length of the injured wrist, which is strongly associated with clinical outcomes [24], was restored from 3.7 mm preoperatively to 11.2 mm at last follow-up. Although the effects of shortened radial length are widely known, the effects of overlengthening have never, to our knowledge, been reported. Although the prognosis of wrists with overlengthening has not been determined, none of the patients in the present study experienced any complications related to the procedure until final follow-up.

**Fig. 5:**
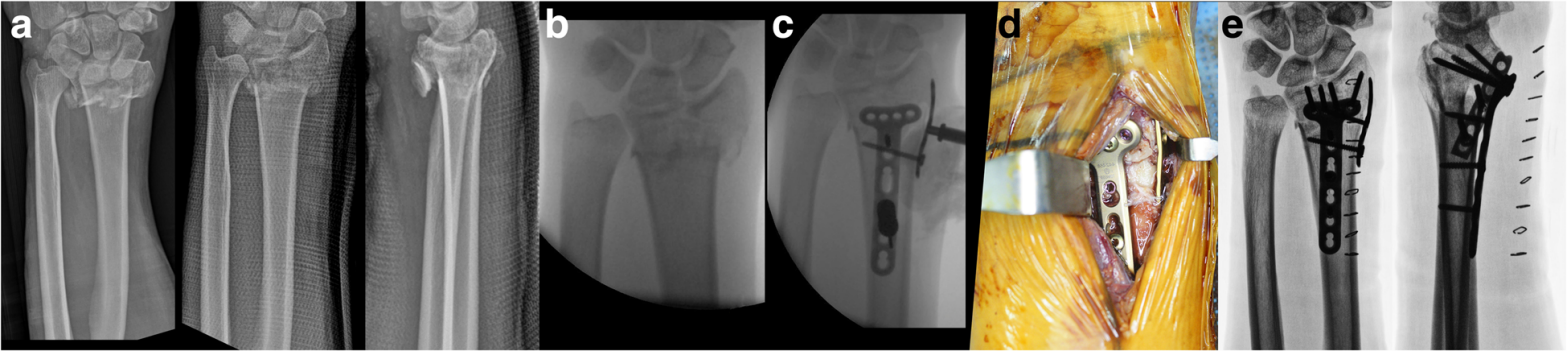
**a** Plain radiographs showing a metaphyseal comminuted fracture of the distal radius with radial length shortening. **b** Despite manipulations of the injured wrist under anesthesia, articular congruity was not restored because the radial styloid fragment was not reduced. **c** Use of the conventional flexor carpi radialis approach by splitting the PQ muscle from its origin to buttress the radial styloid fragment with the plate. **d** Orthogonal plate fixation of the distal radius through the conventional approach. **e** Postoperative radiographs showed the restoration of articular congruity and radial length

Following the introduction of the 2.4 mm volar locking plate system, five locking screws could be inserted into the subchondral row of the distal fragment [1]. Thus, indications for this procedure may be expanded to include extra-articular fractures with metaphyseal comminutions in both cortices, regardless of the size of the distal fragment (Fig. 4). Single volar plating of an articular fracture requires the articular fragment to be of sufficient size, large enough for at least two screws of the 2.4 mm locking plate system [25]. We found that, following satisfactory reduction of the articular surface, the indications were similar for bridge plating and conventional options in the treatment of articular fractures. However, it is very important to verify the reduction status of the radial styloid fragment, which is closely related to articular step-off and radial inclination. Thus, single volar plate fixation using the PQ-sparing approach could be performed if the radial styloid fragment of the articular fracture was sufficiently large for the fixation of two or three screws and if articular congruity was restored by indirect reduction and percutaneous maneuvers. However, if reduction cannot be obtained, it is better to switch to ORIF (Fig. 5).

Our experience clearly demonstrated that the term biologic fixation through bridge plating (BFBP) is more accurate than MIPO, since the two incisions might be larger in size than those of the conventional technique. In addition, this retrospective analysis indicates that BFBP has several potential limitations. First, the use of two incisions may lead to cosmetic problems, especially in younger patients. Second, the operation time was longer and radiation exposure was greater with BFBP than with the conventional technique. Third, the PQ-sparing technique was not compared directly with the conventional technique [20]. Nevertheless, our study, as well as others [19–21], reported satisfactory outcomes with the PQ-sparing technique, suggesting that the latter may reduce postoperative pain, enable motion exercises to be started sooner after surgery, and maintain the muscular envelope of the distal radius, thus potentially avoiding postoperative complications. As this study was not adequately powered due to the lack of a direct comparison group, further studies are required to optimize plate removal and the degree of radiation exposure, as well as to directly compare the PQ-sparing and conventional techniques.

## Conclusions

A combination of bridge plating through a PQ-sparing approach and the distractive technique results in good reduction, with restoration of radial length and reasonable long-term functional outcomes. Compared with the conventional technique, the PQ-sparing technique, despite disadvantages such as technical difficulties, increased radiation exposure, and cosmetic problems, may be helpful in managing metaphyseal comminuted fractures of both cortices with reduced RUI.

## Abbreviations

BFBP: Biologic fixation through bridge plating
FCR: Flexor carpi radialis
GA: Gustilo-Anderson
MGWS: Modified Gartland-Werley Score
MIPO: Minimally invasive plate osteosynthesis
MMWS: Modified Mayo Wrist Score
PACS: Picture archiving and communication system
PQ: Pronator quadratus
RUI: Radio-ulnar index
